# Supplementary material for: Beacon Reconstruction Attack: Reconstruction of genomes in genomic data-sharing beacons using summary statistics
Source: Bioinformatics. 2025 May 19;41(6):btaf273. doi: 10.1093/bioinformatics/btaf273 (PMC12133290; doi:10.1093/bioinformatics/btaf273)
Supplement: btaf273_Supplementary_Data [file btaf273_supplementary_data.zip › Beacon_Reconstruction_supplement_Bioinformatics[AU].pdf]

Supplementary Material
for
Beacon Reconstruction Attack: Reconstruction of genomes in genomic data-sharing beacons using summary statistics

1 Supplementary Notes

1.1 Effect of already reidentified individuals

Here, we investigate the effect of the attacker having access to the genomes of a percentage of the beacon participants through data leaks and reidentification attacks as discussed in Section **??**. We randomly select $p = 20\%$ and 40% of the individuals in both data sets and assume that their genomes are leaked to the attacker who has reidentified them as participants in the beacon dataset. The attacker fixes these individuals in $\mathbf{B}'$ and reconstructs the rest $(1 - p)$ of the individuals for varying numbers of SNPs $|M'|$ and beacon sizes N using the optimization-based method. To observe the effect of individuals already reidentified in reconstruction performance, we reconstruct the remaining $(1 - p)$ of the individuals in the beacon using the same method, but without the assumption of the attacker having access to the genomes of any person in the beacon and compare the results. This is indicated as "Without known p" in Supplementary Figure S11.

We observe that having access to a portion of the beacon leads to higher performance for both $p = 20\%$ and 40% compared to reconstructing the same number of individuals but without using the information of known 20% and 40%. The improvement in F1-score is on average 5.59%. The larger p leads to better reconstruction performance as the search space is smaller for the optimizer. The F1-score gap between $p = 20\%$ and 40% is on average 3.2%. For a beacon with 50 individuals, if the attacker has access to only 10 genomes, the reconstruction achieves an F1-score of 70.7% which makes the attack even stronger, further underscoring the importance of the threat. The same experiment with varying SNP set sizes can be found in Supplementary Figure 3 for the HapMap dataset where we observe the same trend.

1.2 Time Performance

Supplementary Figure 6 shows the time required to reconstruct the beacons with varying N and $|M'|$. The optimization-based algorithm scales linearly with N and $|M'|$. With $N = 100$ and $|M'| = 2000$, the attack takes around 10 hours which was the most time-consuming case in our analyses. The attack is performed offline and the results show that it can scale for large numbers of variants and beacon sizes.

2 Supplementary Figures

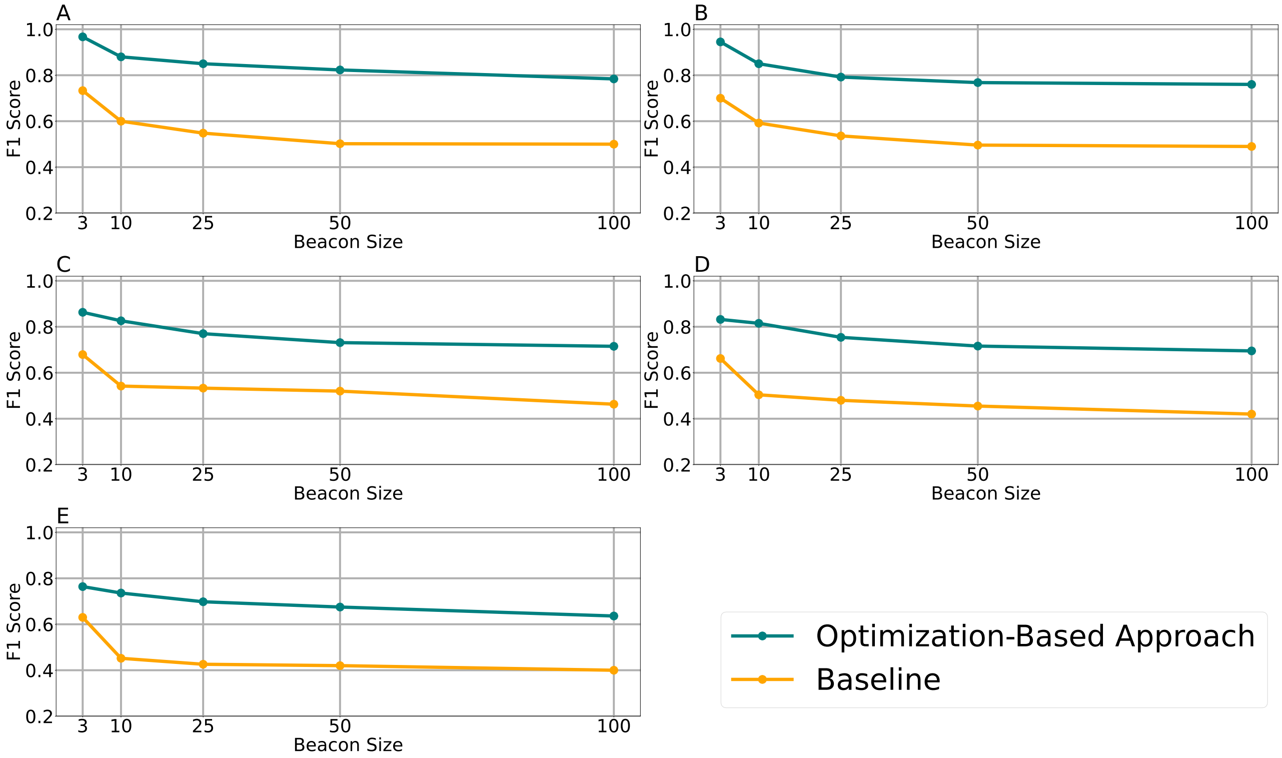

Supplementary Figure S1: F1-Score comparison across different ($|M'| = 30, 50, 100, 500, 2000$) and varying numbers of individuals in the OpenSNP dataset. Plots A–E correspond to these $|M'|$, respectively.

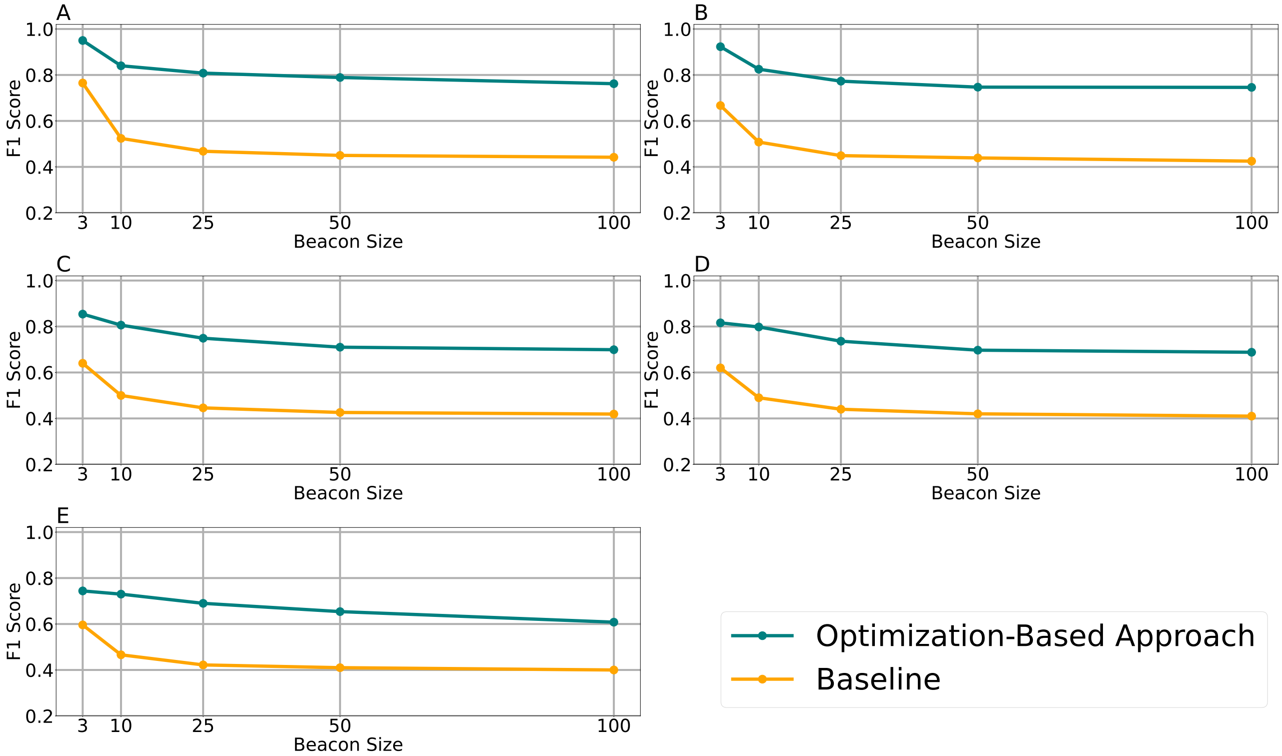

Supplementary Figure S2: F1-Score comparison across different ($|M'|$ = 30, 50, 100, 500, 2000) and varying numbers of individuals in the HapMap dataset. Plots A–E correspond to these $|M'|$, respectively.

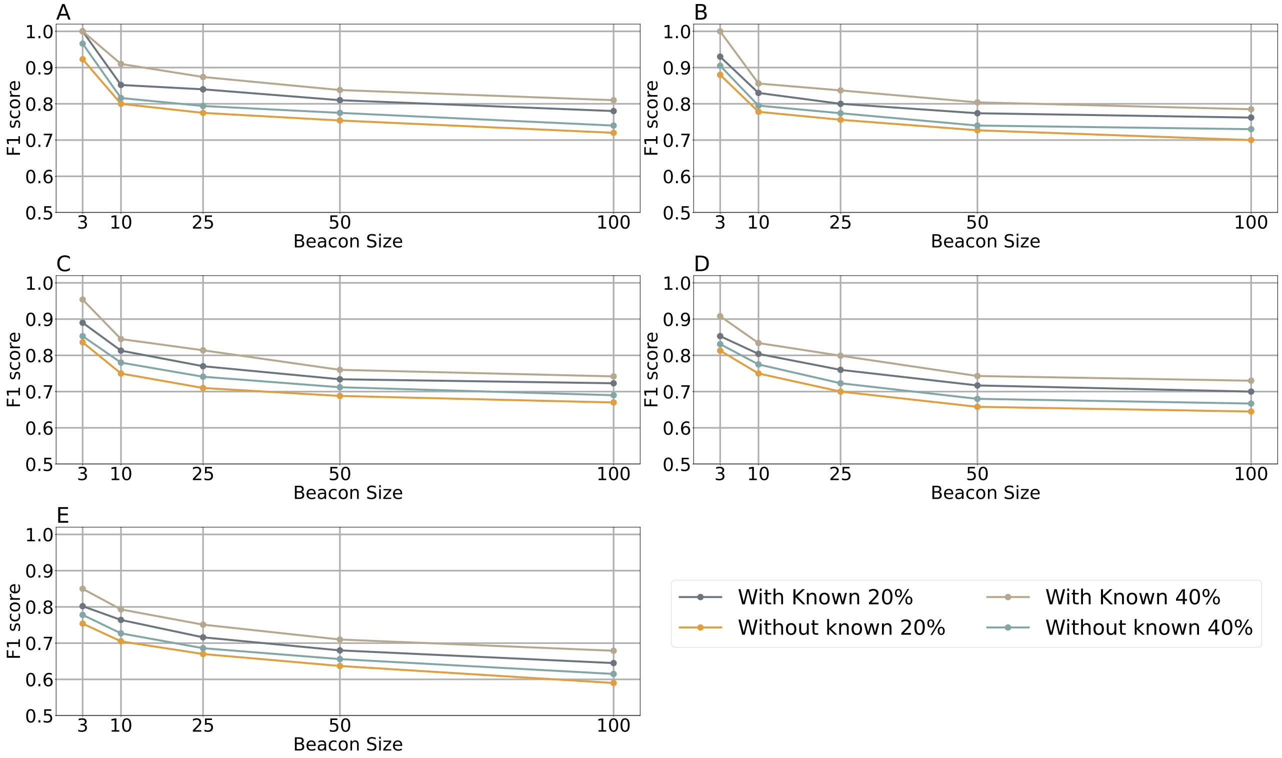

Supplementary Figure S3: F1-Score comparison across different ($|M'|$ = 30, 50, 100, 500, 2000) and varying numbers of individuals in the HapMap dataset. Plots A–E correspond to these $|M'|$, respectively. The results compare the performance of the attacker with access to genomes of p = 20% ("With Known 20%") and p = 40% ("With Known 40%") of participants, and without this information ("Without known 20%" and "Without known 40%").

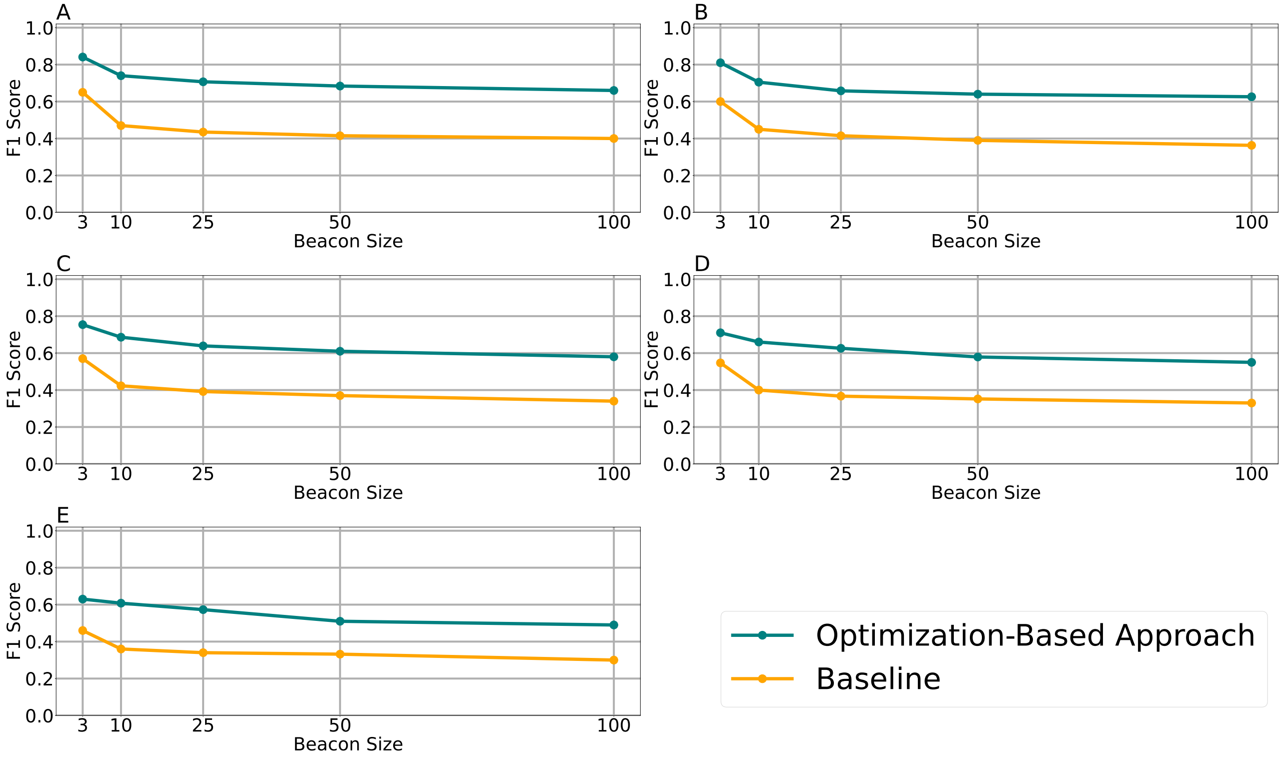

Supplementary Figure S4: F1-Score comparison using MAFs of 64 left-out individuals out of 164 of HapMap dataset. The comparison is across different ($|M'| = 30$, 50, 100, 500, 2000) and varying numbers of individuals. Plots A–E correspond to these $|M'|$, respectively.

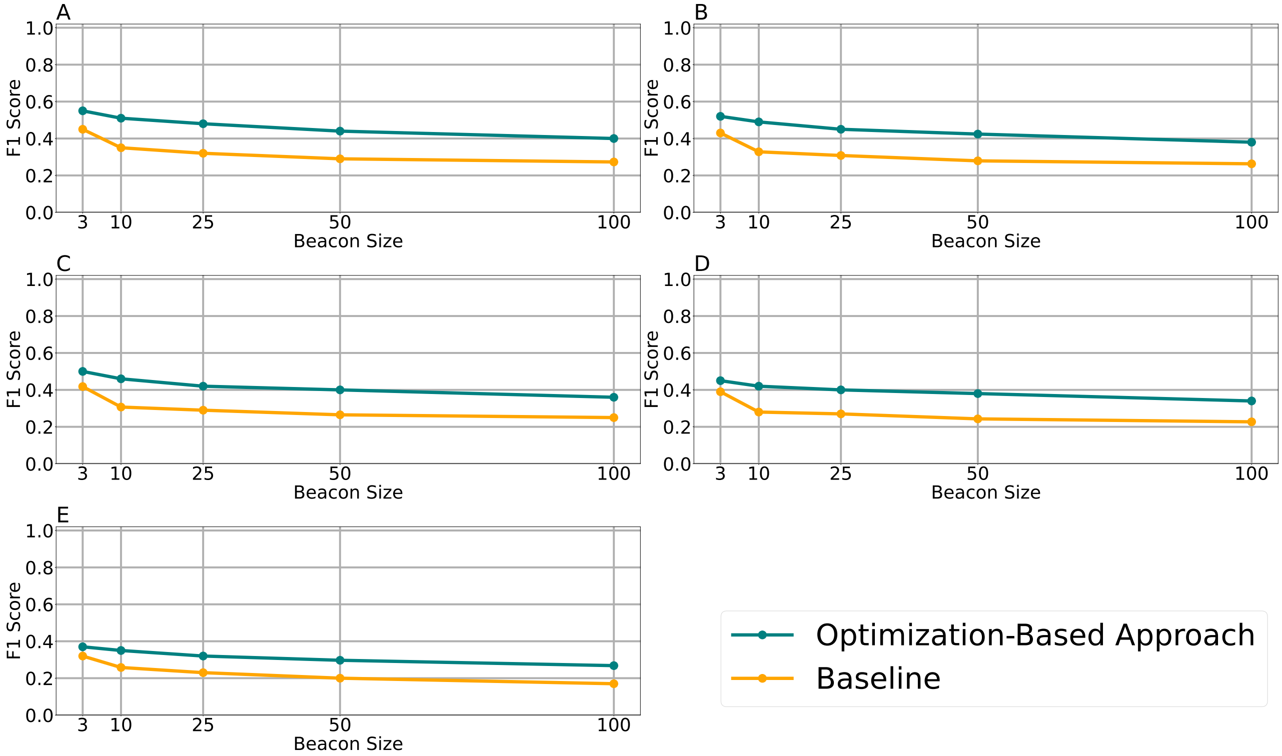

Supplementary Figure S5: F1-Score comparison for the reconstruction of the HapMap dataset using Mexican population MAFs, evaluated across varying numbers of individuals and ($|M'|$ = 30, 50, 100, 500, 2000). Plots A–E correspond to these ($|M'|$, respectively.

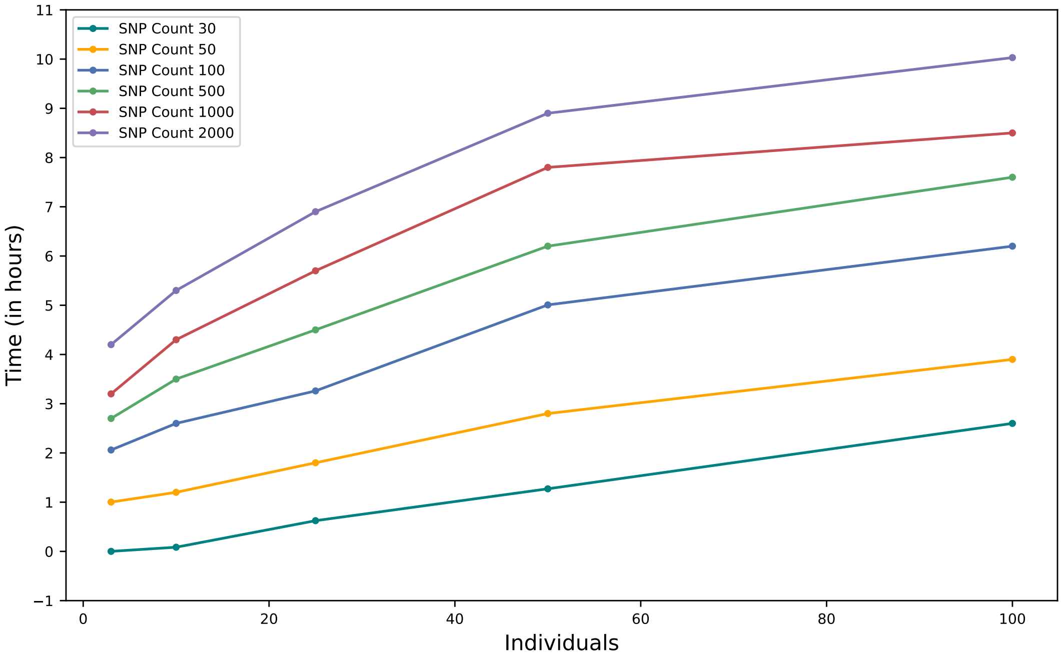

Supplementary Figure S6: Time Analysis Graph across ($|M'|$ = 30, 50, 100, 500, 2000) and varying numbers of individuals

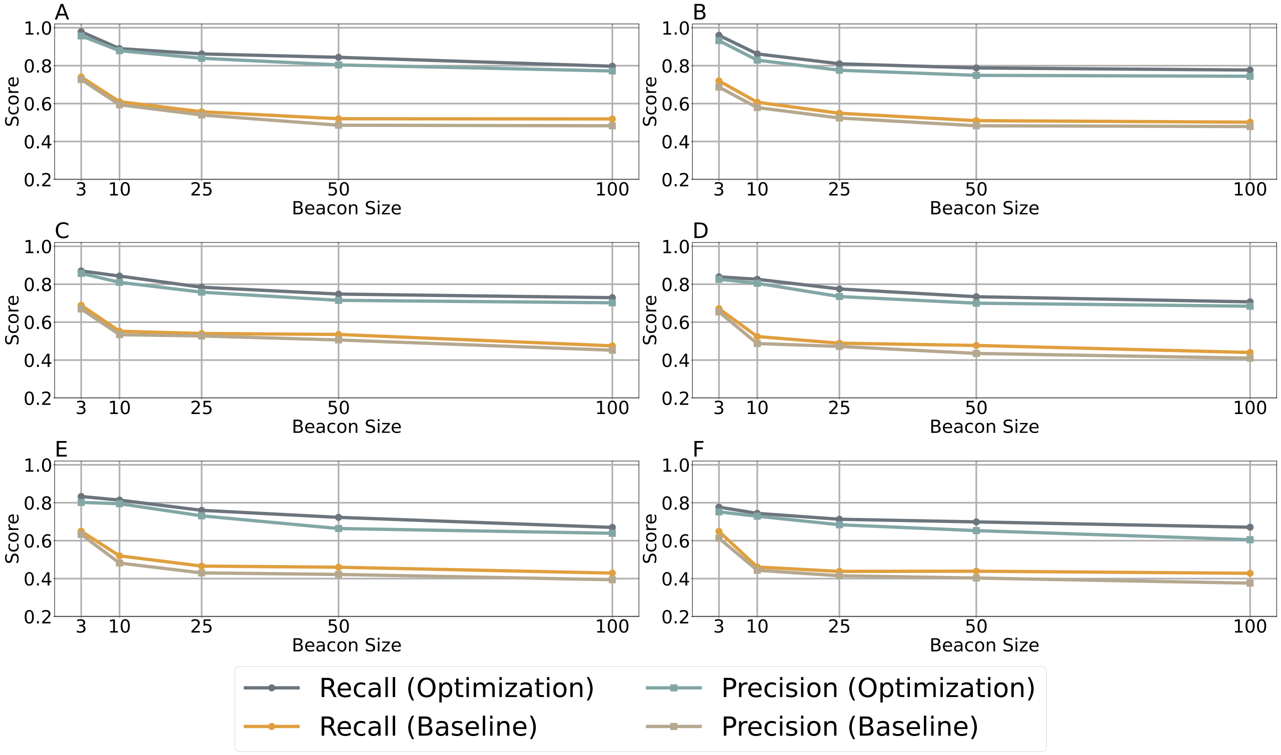

Supplementary Figure S7: Precision and Recall comparison for the reconstruction of the OpenSNP dataset evaluated across varying numbers of individuals and ($|M'|$ = 30, 50, 100, 500, 1000 and 2000). Plots A–F correspond to these ($|M'|$, respectively.

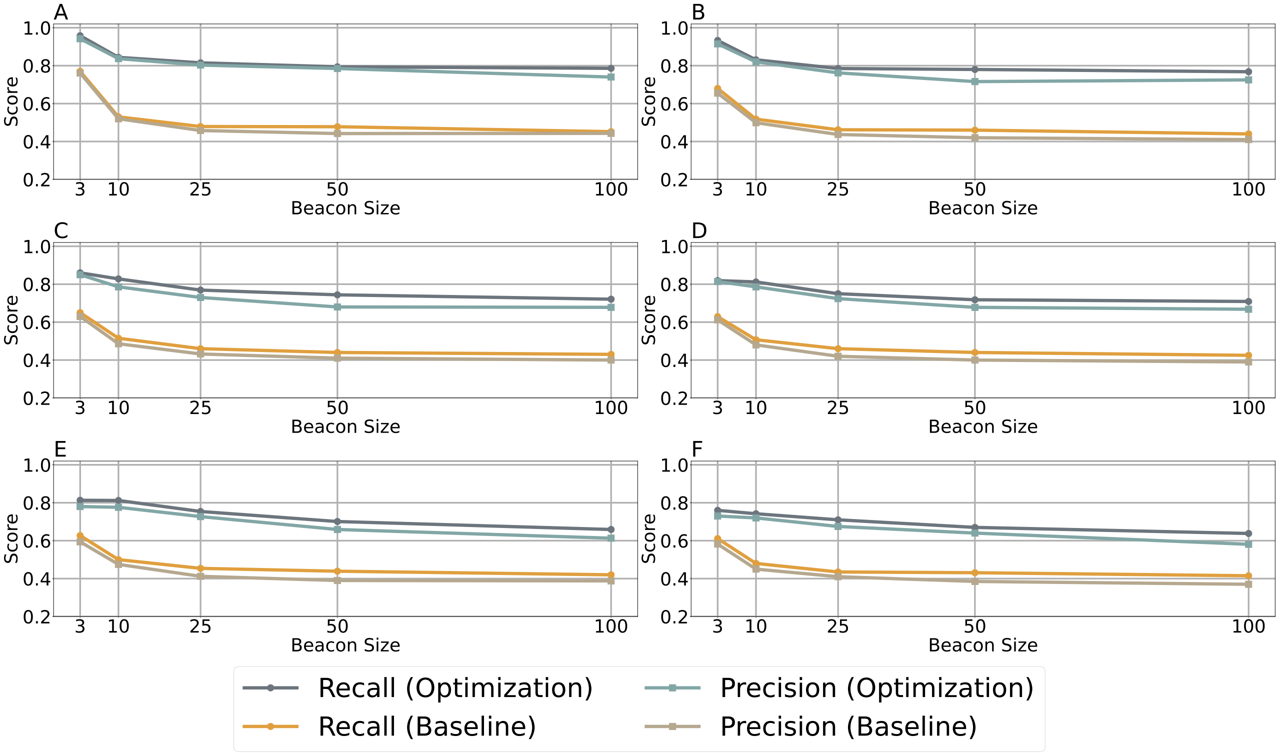

Supplementary Figure S8: Precision and Recall comparison for the reconstruction of the HapMap dataset evaluated across varying numbers of individuals and ($|M'| = 30$, 50, 100, 500, 1000 and 2000). Plots A–F correspond to these ($|M'|$, respectively.

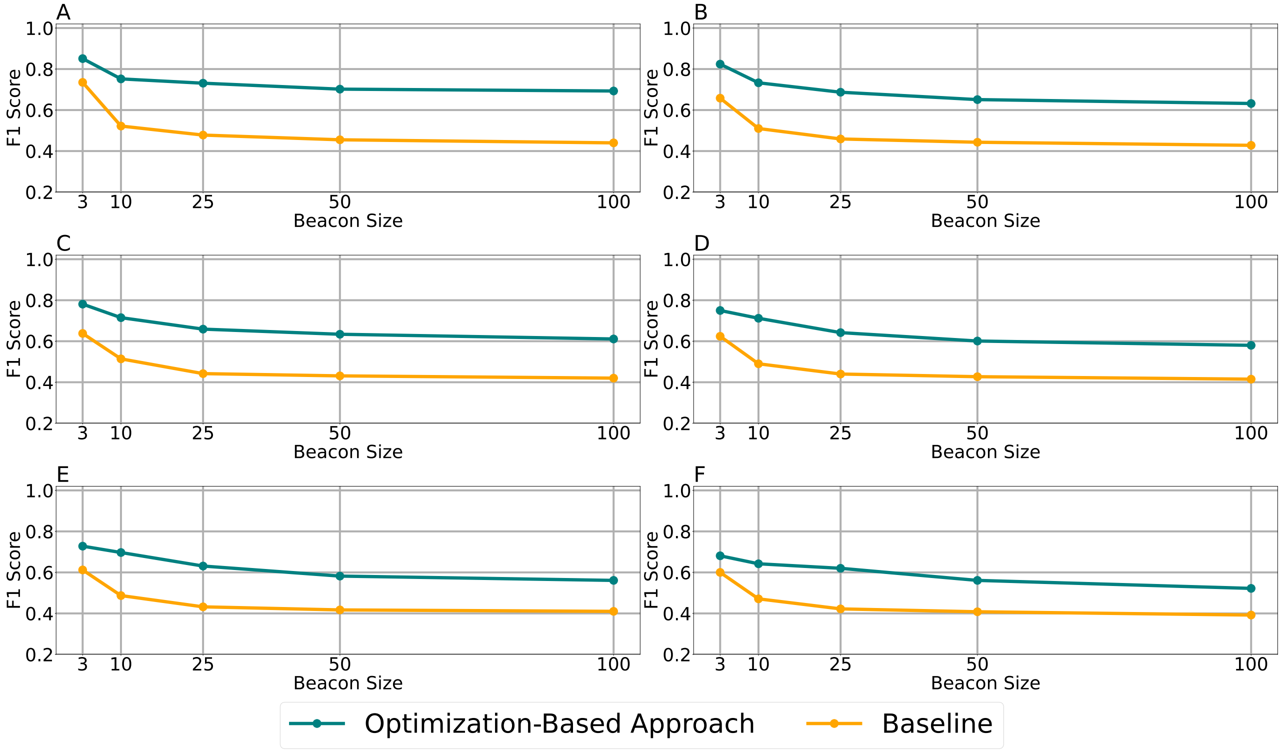

Supplementary Figure S9: F1-Score comparison across different ($|M'| = 30, 50, 100, 500, 1000, 2000$) and varying numbers of individuals in the Gujarati Indians in Houston, TX, USA [GIH] dataset. Plots A–F correspond to these $|M'|$, respectively.

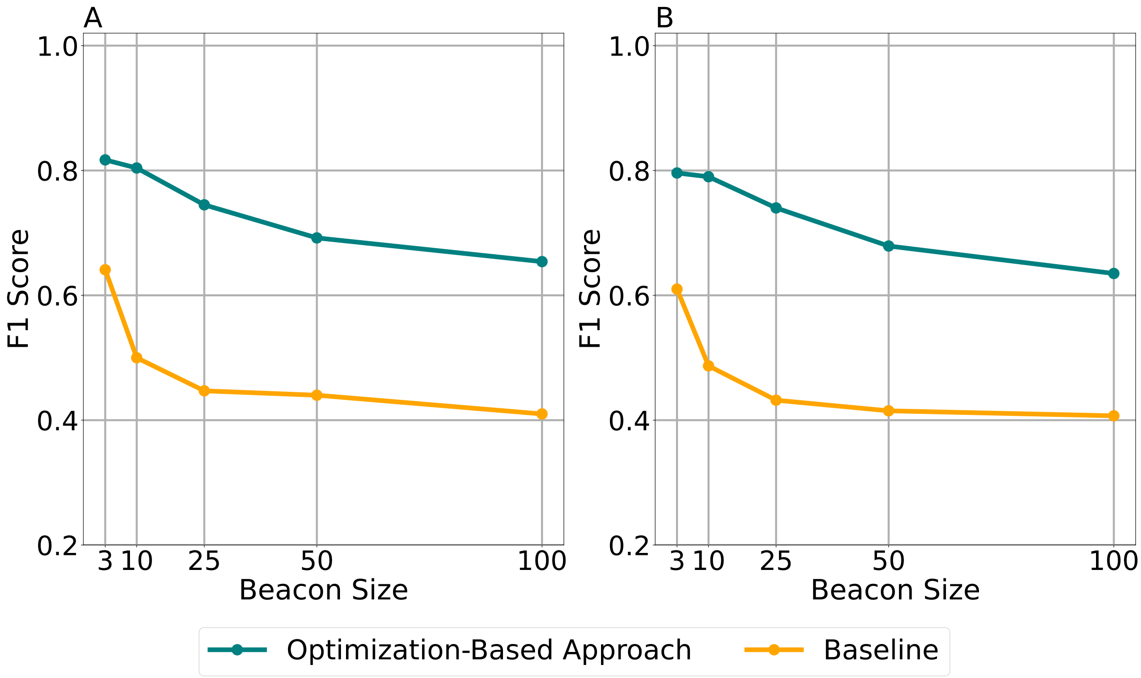

Supplementary Figure S10: F1-Score comparison for $|M'| = 1000$ for varying beacon size (N). Plot A represents the reconstruction of the OpenSNP-based beacons, Plot B represents the reconstructions of the HapMap-based beacons.

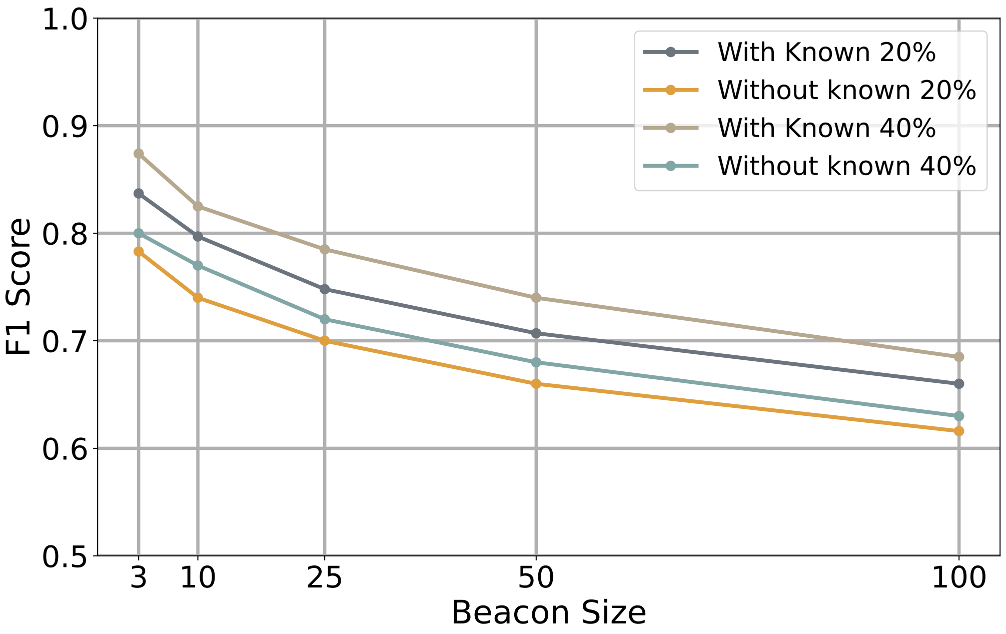

Supplementary Figure S11: F1-Score comparison for $|M'| = 1000$ in the HapMap dataset for varying beacon size (N). The results compare the performance of the attacker to reconstruct same number of genomes with access to genomes of $p = 20\%$ ("With Known 20%") and $p = 40\%$ ("With Known 40%") of participants, and without this information ("Without known 20%" and "Without known 40%").
